# Current understanding and future perspectives on the impact of changing NAFLD to MAFLD on global epidemiology and clinical outcomes

**DOI:** 10.1007/s12072-023-10568-z

**Published:** 2023-08-09

**Authors:** Karl Vaz, Daniel Clayton-Chubb, Ammar Majeed, John Lubel, David Simmons, William Kemp, Stuart K. Roberts

**Affiliations:** 1 Department of Gastroenterology and Hepatology, Ground Floor Alfred Centre, Alfred Health, 55 Commercial Road, Melbourne, VIC 3004 Australia; 2 Central Clinical School, Monash University, Melbourne, Australia; 3 Macarthur Clinical School, School of Medicine, Western Sydney University, Campbelltown, Australia

**Keywords:** NAFLD, MAFLD, Epidemiology, Prevalence, Mortality, Morbidity, Cardiovascular disease, Liver-related outcome, Cancer, Hepatocellular carcinoma

## Abstract

**Introduction:**

For the first time in nearly half a century, fatty liver disease has undergone a change in name and definition, from the exclusive term, non-alcoholic fatty liver disease (NAFLD), to the inclusion-based, metabolic-associated fatty liver disease (MAFLD). This has led investigators across the globe to evaluate the impact the nomenclature change has had on the epidemiology and natural history of the disease.

**Methods:**

This systematic review provides a comprehensive overview on how the shift in name and diagnostic criteria has influenced point prevalence in different geographic regions, as well as morbidity and mortality risk, whilst highlighting gaps in the literature that need to be addressed.

**Conclusions:**

MAFLD prevalence is higher than NAFLD prevalence, carries a higher risk of overall mortality, with greater granularity in risk-stratification amongst MAFLD subtypes.

**Graphical abstract:**

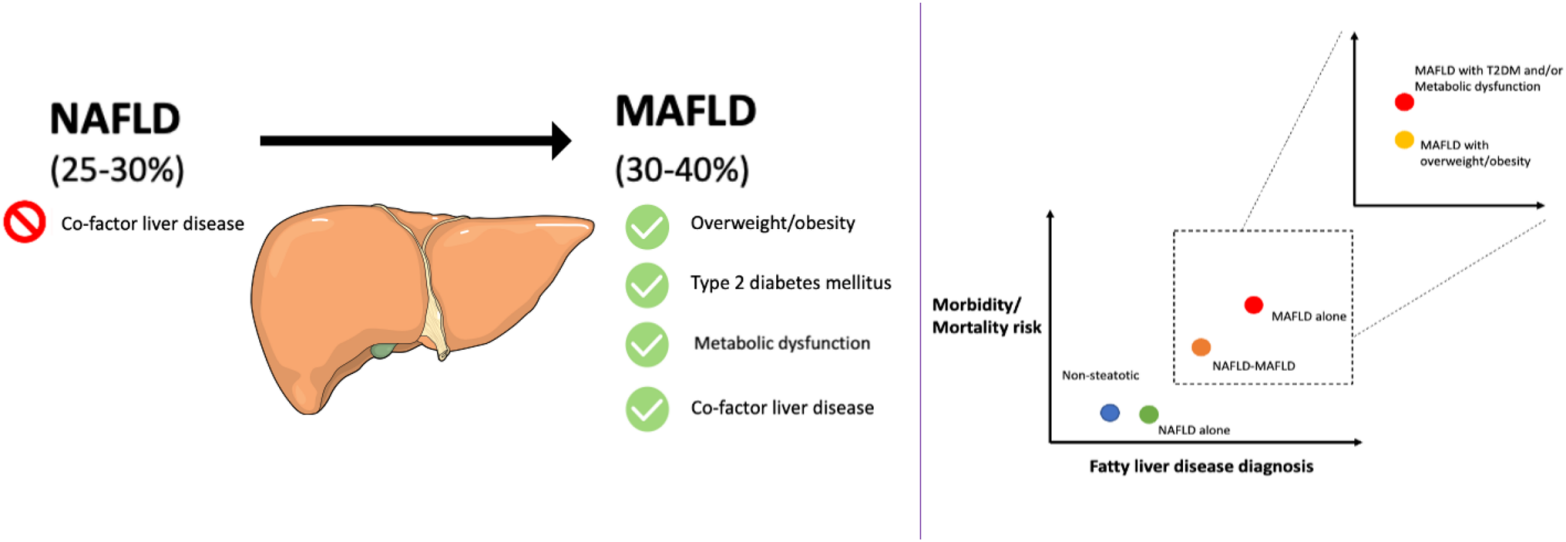

**Supplementary Information:**

The online version contains supplementary material available at 10.1007/s12072-023-10568-z.

## Introduction

Non-alcoholic steatohepatitis (NASH) first entered the hepatology vernacular in 1980, when Ludwig and colleagues described the histologic finding of fatty change with lobular inflammation resembling alcoholic hepatitis in 20 patients who did not consume alcohol and in whom there was no alternate cause of liver disease [1]. Understanding of the condition has expanded exponentially over the past 4 decades as worldwide non-alcoholic fatty liver disease (NAFLD) has become the most prevalent condition affecting the liver, mirroring the burgeoning obesity pandemic [2] and rapidly emerging as one of the foremost indications for liver transplantation [3, 4].

In 2020, an international consensus panel—comprising select experts in the field from 22 countries across the globe—revisited the nomenclature and posited the term metabolic dysfunction-associated fatty liver disease, or simply metabolic-associated fatty liver disease (MAFLD) [5, 6]. This newly proposed term endeavors to better encapsulate the pathophysiological basis for the condition, remove stigmatizing terminology, and acknowledge the heterogeneity encountered in clinical practice, with reference to the co-existence of multiple etiologies of liver disease in a single patient. Moreover, a more inclusive diagnostic criteria may positively influence enrollment into clinical trials and highlight with greater precision the synergistic impact on clinical outcomes.

For both NAFLD and MAFLD, evidence of ≥ 5% hepatosteatosis is a sine qua non for diagnosis irrespective of detection modality. Whereas NAFLD is reliant on excluding alternate causes of liver disease (i.e., alcohol-related fatty liver, viral hepatitis, and drug-induced steatosis) [7, 8], MAFLD requires at least one of the following to be present: (1) overweight according to body mass index (specific threshold for those of Asian ethnicity versus other ethnicities); (2) type 2 diabetes mellitus (T2DM) as per standard diagnostic criteria; and/or (3) metabolic ‘dysfunction’ defined by presence of at least two of seven clinical and biochemical criteria [5] (Table 1).

**Table 1:** Differences in diagnostic criteria between MAFLD and NAFLD and rationale for each

MAFLD	NAFLD	Rationale
≥ 5% hepatosteatosis with; 1. Overweight—BMI ≥ 23 kg/m^2^ (Asian population) or ≥ 25 kg/m^2^ (all other ethnicities) 2. T2DM—per standard diagnostic criteria 3. Metabolic dysfunction—any ≥ 2 of; (i) Elevated waist circumference: ≥ 90 cm/80 cm (males/females) among Asian population or ≥ 102 cm/88 cm (males/females) among all other ethnicities (ii) Blood pressure ≥ 130/85 mmHg or need for antihypertensive therapy (iii) Plasma triglycerides ≥ 1.70 mmol/L or need for specific lipid-lowering therapy (iv) Plasma HDL-cholesterol < 1.0 mmol/L for males or < 1.3 mmol/L for females or need for specific therapy (v) Prediabetes according to standardized criteria (vi) HOMA-IR score ≥ 2.5 (vii) Plasma HS-CRP > 2 mg/L	≥ 5% hepatosteatosis without any other etiology of liver disease	For MAFLD; • Name change and diagnostic criteria better encapsulate pathogenesis of disease, namely metabolic dysregulation and insulin-resistance • Inclusive criteria allowing for recognition of co-factors for liver disease which may impact additively or synergistically on natural history and clinical outcomes. This better reflects heterogeneity seen in clinical practice and can positively impact drug trial recruitment • Cut-off for alcohol consumption to discriminate ‘safe’ from ‘excessive’ with regard to steatogenic and fibrogenic potential not well established • Removes potentially stigmatizing and trivial terms (i.e., ‘alcoholic’ and ‘non-’) • May lead to greater disease recognition among health professionals beyond hepatology For NAFLD; • Concern around impact on stakeholder acceptance, especially industry and regulatory bodies with impact on drug and biomarker discovery, development and acceptance (particularly with currently accepted histologic outcome measures for drug-development) • Uncertainty around what entails ‘metabolic health’ and hence around criterion three (metabolic dysfunction) of proposed diagnostic criteria • Lack of consensus among major hepatological societies

*MAFLD* metabolic-associated fatty liver disease, *NAFLD* non-alcoholic fatty liver disease, *BMI* body mass index, *HDL* high-density lipoprotein, *HOMA-IR* homeostatic model assessment for insulin resistance, *HS-CRP* high-sensitivity C-reactive protein

However, a debate has since ensued on the world stage with several leading commentators voicing concerns over the timing of the name change and proposed criteria for diagnosis. Criticisms include reference to the operational definition for ‘metabolic health’, the risk of confusing colleagues outside the discipline whereby disease awareness remains substandard, as well as the potential for unintended negative consequences on the clinical development and regulatory approval pathways of novel therapeutics [9–11]. As such, of the three major international hepatology societies, only the Asian Pacific Association for the Study of the Liver (APASL) has officially endorsed the paradigm shift [12], with observers eagerly awaiting consensus statements from European Association for the Study of the Liver (EASL) and American Association for the Study of Liver Diseases (AASLD). EASL and AASLD have undertaken a joint formal Delphi process to address the merits of adopting the name change in fatty liver disease [13, 14], with the consensus yet to be published at the time of this review. A particular focus has been on ensuring the shift from NAFLD to MAFLD does not inadvertently impact stakeholder enthusiasm around drug and biomarker development.

Controversies in nosology aside, the dawn of MAFLD has brought with it fertile ground for research, with further opportunities for investigation remaining on the horizon. Herein, we look to summarize in the form of a narrative review, the literature on the influence the name change from NAFLD to MAFLD has had on epidemiology and clinical outcomes in adults and provide key areas for future research efforts.

## Methodology

A search of OVID Medline, EMBASE and Web of Science databases from inception to May 2023 was carried out to identify studies reporting on the differences in prevalence and clinically relevant outcomes for NAFLD and MAFLD in the same cohort (Supplementary Fig. 1). The search terms included NAFLD and MAFLD and their associated terms. Studies reporting prevalence as a secondary outcome whereby the primary outcome was to determine an association between fatty liver disease and one more clinicobiochemical parameter(s) were excluded if the study exclusion criteria led to sampling and/or ascertainment bias.

## Prevalence

Given the geographic variation in acceptance of the new term MAFLD, it is not surprising that prevalence studies have been most represented by those conducted in Asia compared to Europe, North America and other regions as detailed below (Table 2).

**Table 2:** Summary of cross-sectional studies reporting on point prevalence difference between NAFLD and MAFLD

Study	Country	Year	Number of participants	Age and gender profile	Detection method	NAFLD (%)	MAFLD (%)	Concordance (%)	Concomitant liver disease
North America									
Lin et al. 2020 (15)	USA (NHANES III)	1988–1994	13,083	Adults ≥ 20 years old Mean age 44 ± 16 47% male	Ultrasound	33.2	31.2	–	ARLD 8.4%
Zhang et al. 2023 [16]	USA (NHANES III)	1988–1994	11,673	Adults 20 to 79 years old 47% male	Ultrasound	33.2	30.5	–	–
Huang et al. 2021 [17]	USA (NHANES III)	1988–1994	12,480	20–74 years old Mean age 42 49% male	Ultrasound	30.3	31.3	73.3 14.8% MAFLD-only 11.9% NAFLD-only	ARLD 19.8% Viral 2.9%
Nguyen et al. 2021 [22]	USA (NHANES III)	1988–1994	13,640	≥ 20 years old	Ultrasound	18.3	20.1	74.7 16.8% MAFLD-only 8.5% NAFLD-only	ARLD 9.3%
Aimuzi et al. 2023 [23]	USA (NHANES)	2011–2018	2618	≥ 20 years old 49% male	US-FLI	29.1	32.7	–	–
Ciardullo et al. 2021 [24]	USA (NHANES)	2017–2018	1710	All adults Mean age 46 49% male	VCTE	37.1	39.1	90.8 7.4% MAFLD-only 1.8% NAFLD-only	ARLD 5.3% HCV 2.0% HBV 0.3%
Xie et al. 2022 [25]	USA (NHANES)	2017–2018	4494	All adults Mean age 47 ± 17 49% male	VCTE	37.2	48.0	–	–
Wong et al. 2022 [26]	USA (NHANES)	2011–2018	-	All adults	US-FLI	–	34.8 34.4 in 2011–12 38.1 in 2017–18	–	ARLD 5.5% HCV 1.6% CHB 0.5%
Zhang et al. 2021 [27]	USA (NHANES)	1999–2016	19,617	≥ 20 years old	US-FLI	26.4 in 1999–2002 33.0 in 2011–2016	28.4 in 1999–2002 35.8 in 2011–2016	–	–
Asia									
Xu et al. 2023 [28]	China (Jiangsu—Nanjing Medical University)	2014–2015	72,392	All adults	Ultrasound	31.5	28.3	87.8 1.1% MAFLD-only 11.2% NAFLD-only	0.4% ARLD
Wang et al. 2022 [29]	China (Tangshan city—Kailuan Study)	2006–2012	152,139	All adults 81% male	Ultrasound	27.3	31.5	83.2 15.0% MAFLD-only 1.8% NAFLD-only	ARLD 13.4% CHB 2.2%
Liang et al. 2022 [30]	China (Shanghai—Nicheng Cohort Study)	2013–2014	6873	45–70 years old Median 62, IQR 59–65 42% male	Ultrasound	40.3	46.7	–	ARLD 10.0% CHB 4.8%
Yu et al. 2022 [31]	China (Jinchang city)	2011–2013	30,633	All adults Mean 46 ± 13 64% male	Ultrasound	18.8	21.0	78.8 15.5% MAFLD-only 5.7% NAFLD-only	–
Liu et al. 2022 [32]	China (Shanghai)	2020	795	> 20 years old Mean age 45 ± 10 61% male	Ultrasound	43.4	44.8	90.5 6.3% MAFLD-only 3.3% NAFLD-only	–
Miao et al. 2022 [33]	China (four cities in Central and Southeast)	2016–2020	2543	≥ 40 yo 61% male	Ultrasound	18.4	20.4	–	–
Wang et al. 2022 [34]	China (SPECT-China)	2014	12,183	All adults 41% male	Ultrasound	45.3	48.4	86.3 9.9% MAFLD-only 3.8% NAFLD-only	Any 10.3%
Yuan et al. 2022 [35]	China (Tangshan city—Kailuan Study)	2006–2014	151,391	All adults 81% male	Ultrasound	26.0	31.4	78.8 19.3% MAFLD-only 1.9% NAFLD-only	ARLD 17.6% CHB 2.2%
Zeng et al. 2022 [36]	China (REACTION Cohort)	2011–2012	9927	≥ 40 years old Mean age 56 ± 8 33% male	Ultrasound	36.9	40.3	83.2 12.5% MAFLD-only 4.3% NAFLD-only	–
Wong et al. 2021 [37]	China (Hong Kong)	2008–2010	1013	All adults Mean 48 ± 10 43% male	H-MRS	25.7	25.9	89.2 5.8% MAFLD-only 5.1% NAFLD-only	CHB 4.9% ARLD 1.1%
Cheng et al. 2023 [38]	Taiwan (Taiwan Biobank)	2008–2022	22,909	> 20 years old	Ultrasound	36.9	38.9	79.7 11.8% MAFLD-only 7.0% NAFLD-only	CHB 8.5% ARLD 2.7% HCV 2.1%
Lee et al. 2021 [39]	Korea (National Health Insurance Service)	2009–2010	9,584,399	40–64 years old Median 50 49% male	FLI	28.0	37.3	72.4 26.1% MAFLD-only 1.5% NAFLD-only	ARLD 22.2% Viral 4.6% Other 1.5%
Chun et al. 2022 [40]	Korea (Seoul—Severance Health Checkup)	2014–2019	78,762	Mean age 49 ± 12 56% male	Ultrasound	30.5	34.3	78.4 16.1% MAFLD-only 5.5% NAFLD-only	ARLD 12.6%
Choi et al. 2022 [41]	Korea (Seoul National University Hospital Healthcare System Gangnam)	2013–2017	3195	Mean age 55 69% male	Ultrasound	34.6	46.8	–	–
Kim et al. 2022 [42]	Korea (Seoul—Severance Health Checkup)	2016–2019	2144	All adults Mean age 56 ± 9 63% male	Ultrasound	41.6	46.4	78.9 15.5% MAFLD-only 5.6% NAFLD-only	ARLD 12.8%
Kim et al. 2023 [43]	Korea (Kangbuk Samsung Health Study)	2002–12	394,835	All adults Mean age 40 ± 10 55% male	Ultrasound	22.2	25.0	78.0 16.2% MAFLD-only 5.7% NAFLD-only	Viral 3.5%
Seo et al. 2021 [44]	Korea (Seoul National University Hospital Healthcare System Gangnam)	2012	3441	Mean age 52 62% male	Ultrasound	33.2	32.8	–	–
Yoo et al. 2023 [45]	Korea (Kangbuk Samsung Health Study)	2002–2019	701,664	Mean 40 ± 11 53% male	Ultrasound	22.5	25.3	76.4 16.7% MAFLD-only 6.1% NAFLD-only	ARLD 16.4% HCV 0.1% CHB 1.7%
Fujii et al. 2021 [46]	Japan (MedCity21 Health Examination)	2014–2019	2254	All adults	Ultrasound	27.4	35.0	69.9 24.4% MAFLD-only 4% NAFLD only	–
Bessho et al. 2022 [47]	Japan (Tokyo)	2012–2018	890	Mean age 60 ± 12 67% male	Ultrasound	30.1	43.1	62.2 33.3% MAFLD only 4.5% NAFLD-only	–
Tateda et al. 2022 [48]	Japan (Iwaka Health Promotion Project)	2018	950	≥ 20 years old Median age 52, IQR 38–65 42% male	VCTE	24.6	28.7	65.2 22.9% MAFLD-only 10.3% NAFLD-only	–
Sogabe et al. 2022 [49]	Japan (Shikoku Central Hospital)	2016–2018	11,766	Mean age 52 ± 9 52% male	Ultrasound	27.7	35.5	75.4 22.9% MAFLD-only 1.2% NAFLD-only	–
Tanaka et al. 2023 [50]	Japan (Sapporo—Keijinkai Maruyama Clinic)	2006	13,159	Mean age 48 ± 8 65% male	Ultrasound	32.8	32.3	–	–
Mori et al. 2023 [51]	Japan (Sapporo—Keijinkai Maruyama Clinic)	2006	17,021	All adults Mean age 49 ± 9 64% male	Ultrasound	24.1	32.7	–	–
Niriella et al. 2021 [52]	Sri Lanka (Ragama Health Study)	2007	2985	35–64 years old 45% male	Ultrasound	31.5	33.2	87.7 8.6% MAFLD-only 3.7% NAFLD-only	ARLD 8.9%
Oceania									
Kemp et al. 2022 [53]	Australia (CrossRoads II Cohort)	2016–2018	722	All adults Mean 59 ± 16 45% male	FLI	38.7	47.2	82.5 17.5% MAFLD-only	ARLD 16.9%
Europe									
van Kleef et al. 2022 [54]	Netherlands (Rotterdam Study)	2009–2014	5445	≥ 45 years old Mean 70 ± 9 42% male	Ultrasound	29.5	34.3	80.4 16.6% MAFLD-only 3.0% NAFLD-only	ARLD 15.4% Steatogenic medication 1.9%

*NAFLD* non-alcoholic fatty liver disease, *MAFLD* metabolic-associated fatty liver disease, *NHANES* National Health and Nutrition Survey, USA United States of America, *ARLD* alcohol-related liver disease, *US-FLI* United States Fatty Liver Index, *VCTE* vibration-controlled transient elastography, *HCV* hepatitis C virus, *CHB* chronic hepatitis B, *IQR* interquartile range, H-MRS—proton magnetic resonance spectroscopy

*North America:* Lin et al. provide one of the earliest insights into NAFLD vs MAFLD prevalence, through post-hoc analysis of the third National Health and Nutrition Examination Survey from 1988 to 1994 (NHANES III) in the United States (USA) [15]. Hepatosteatosis was determined through ultrasonography (US) with MAFLD prevalence reported to be 31.2%, while NAFLD prevalence 33.2%. A further two studies utilizing NHANES III report the prevalence of both NAFLD and MAFLD to be similar, between 30 and 33% [16, 17]. Concerns arise with the methodology of these studies given a near 1.5-fold higher NAFLD prevalence from NHANES III than existing literature in the pre-MAFLD era (~ 18–20%) [18–21]. Nguyen et al. conducted an equivalent but seemingly more accurate analysis from NHANES III, with MAFLD prevalence 20.1% and NAFLD prevalence 18.3%, with 74.7% concordance of NAFLD-MAFLD and a greater proportion with non-NAFLD-MAFLD than non-MAFLD-NAFLD (16.8% vs 8.5%) [22]. Despite adoption of the same operational definitions for NAFLD and MAFLD among studies, the differences in prevalence estimates from NHANES III demonstrate the inter-reporter variability in epidemiologic studies in fatty liver disease has not been remedied by the nomenclature change. Contemporary iterations of NHANES validate that MAFLD marginally increases fatty liver disease prevalence in the USA, whether case ascertainment is through the United States Fatty Liver Index (US-FLI) or accepted elastographic parameters on vibration-controlled transient elastography (VCTE) [23–25]. A study by Wong et al. from NHANES 2011–2018 reported that MAFLD prevalence increased from 34.4% to 38.1% (*p* < 0.01) between 2011 to 2018, with 7.6% of MAFLD patients having concomitant liver disease (5.5% alcohol-related, 1.6% hepatitis C, 0.5% chronic hepatitis B [CHB]) [26]. This rise in fatty liver disease is supported by another study by Zhang et al. from NHANES 1999–2016, with both NAFLD and MAFLD prevalence rising in parallel [27] (Table 2).

*Asia:* In mainland China, nine cross-sectional, US-based studies have reported the MAFLD prevalence to be between 20.4 and 48.4%, and in all but one study [28] higher than NAFLD prevalence (18.4–45.3%) [28–36]. The difference is accounted for by the region in which they occurred, age of participants (all adults vs ≥ 40 years old vs 45–70 years old only), epoch of study (ranging from 2006 to 2020) and varying prevalence of central and general obesity and T2DM. Seven of these studies reported the NAFLD-MAFLD concordance to be between 78 and 90% [28, 29, 31, 32, 34–36], with all but one [28] finding non-NAFLD-MAFLD cohort significantly higher than the non-MAFLD-NAFLD proportion. While studies report similar rate of co-existent CHB (between 2 and 5% [29, 30, 35]), rates for simultaneous alcohol-related liver disease (ARLD) is vastly different (from 0.4 to 17.6% [28–30, 35]). Notably, in the study by Wang et al., there was a marked difference in ARLD between females and males with MAFLD (0.5 vs 15.8%, respectively) [29].

In one cross-sectional population-based study of adults from Hong Kong conducted between 2008 and 2010 and utilizing proton magnetic resonance spectroscopy to detect hepatosteatosis, MAFLD prevalence was 25.9% and NAFLD prevalence 25.7%, with an overall concordance of 89.2% (5.8% with MAFLD without NAFLD and 5.1% NAFLD without MAFLD) [37]. One study from the Taiwan Biobank cohort of 22,909 adults who underwent US, MAFLD prevalence was higher than NAFLD (38.9% vs 36.9%) with 79.7% concordance between fatty liver disease definitions and the majority of MAFLD-only cohort due to concomitant viral hepatitis, particularly CHB [38].

A FLI-based study from South Korea among close to 10 million participants aged between 40 and 64 years recruited in 2009–2010, the NAFLD-MAFLD concordance was 72.4% (26.1% MAFLD without NAFLD, 1.5% NAFLD without MAFLD), with point prevalence 37.3% and 28.0% for MAFLD and NAFLD, respectively [39]. In all but one of six smaller studies originating in Korea, the finding that MAFLD prevalence is higher than NAFLD prevalence is once more replicated (MAFLD 25–46% vs NAFLD 22–41%) [40–45]. Akin to the studies reporting from China, the differences in fatty liver prevalence between studies can be accounted for by varying geographic region of sampled cohort, gender and age profile of population studied, and prevalence of metabolic risk factors and co-existent liver disease in each cohort. The Hong Kong and FLI-based Korean studies report similar co-prevalence of viral hepatitis as those from China, 4.9% and 4.6% respectively; however, concomitant ARLD differed substantially, 1.1% in Hong Kong and 22.2% in Korea, despite the same definition for excessive consumption (> 30 g/day in males, > 20 g/day in females). Co-existent ARLD in > 10% of MAFLD-only participants was seen among all Korean studies reporting this outcome and mirrors the prevalence in China (Table 2).

In the earliest report establishing prevalence differences with the shift in fatty liver nomenclature from Japan, Fujii et al. report MAFLD prevalence to be 35.0%, higher than NAFLD prevalence 27.4% and with 69.9% concordance from a health examination registry of over 2000 adults undertaking US between 2014 and 2019 [46]. Although concurrent liver disease in the MAFLD-only cohort was not specified, presence of hepatitis C antibody and alcohol consumption ≥ 60 g/day were exclusion criteria, suggesting it was related to lower degrees of alcohol excess, CHB and/or alternate liver disease. Furthermore, 90% of isolated MAFLD participants were male, markedly disparate compared with the gender distribution among those with overlap NAFLD-MAFLD (69% male) and isolated NAFLD (64% male). In other studies from Japan, the concordance between fatty liver disease definitions is seemingly lower than other parts of the globe, with a higher MAFLD-only cohort than reported elsewhere in Asia or beyond [47–49], suggesting this difference may be driven by a higher prevalence of simultaneous liver disease or greater degree of metabolic dysfunction among Japanese fatty liver disease patients. Unfortunately, alternate etiology of liver disease has not been reported in any of these studies. Once more, all but one study reporting the difference in NAFLD and MAFLD prevalence in Japan revealed a higher prevalence of the latter [46–51] (Table 2). However, the methodology of this study comes into question given other authors determined the prevalence of NAFLD to be much lower in the same cohort studied [51], and as per the NHANES III reports highlights the fraught nature of reporting on fatty liver disease epidemiology.

A single study from Sri Lanka utilizing the well-conducted Ragama Health Study which enrolled those between 35 and 64 years old and utilizing US, the concordance was high at 87.7% (8.6% MAFLD without NAFLD, 3.7% NAFLD without MAFLD) with a MAFLD prevalence of 33.2% higher than NAFLD prevalence 31.5% [52] (Table 2).

*Oceania:* One FLI-based cross-sectional study from a regional center in Australia revealed concordance in the two diagnoses was 82.5% (17.5% MAFLD without NAFLD, 0% NAFLD without MAFLD), with a MAFLD prevalence of 47.2% again higher than NAFLD prevalence 38.7% [53]. Co-existent alcohol-related fatty liver was 16.9% in the Australian MAFLD cohort compared to 8.9% in the Sri Lankan cohort, despite lower threshold used to determine excessive alcohol consumption in the Sri Lankan study. Neither study reported on prevalence of viral hepatitis (Table 2).

*Europe:* To date, only one study has been published from Europe, specifically from the Rotterdam study in the Netherlands, recruiting adults older than 45 years between 2009 and 2014 and undertaking US [54]. NAFLD and MAFLD prevalence were 29.5% and 34.3%, respectively, with 80.4% concordance (Table 2). However, there is some uncertainty in the results from this study given participants consuming greater than 60 g of alcohol per day and those with viral hepatitis were excluded, such that concomitant liver disease in the MAFLD participants was related to moderate alcohol excess and use of steatogenic medications.

## Epidemiology

**Fig. 1:**
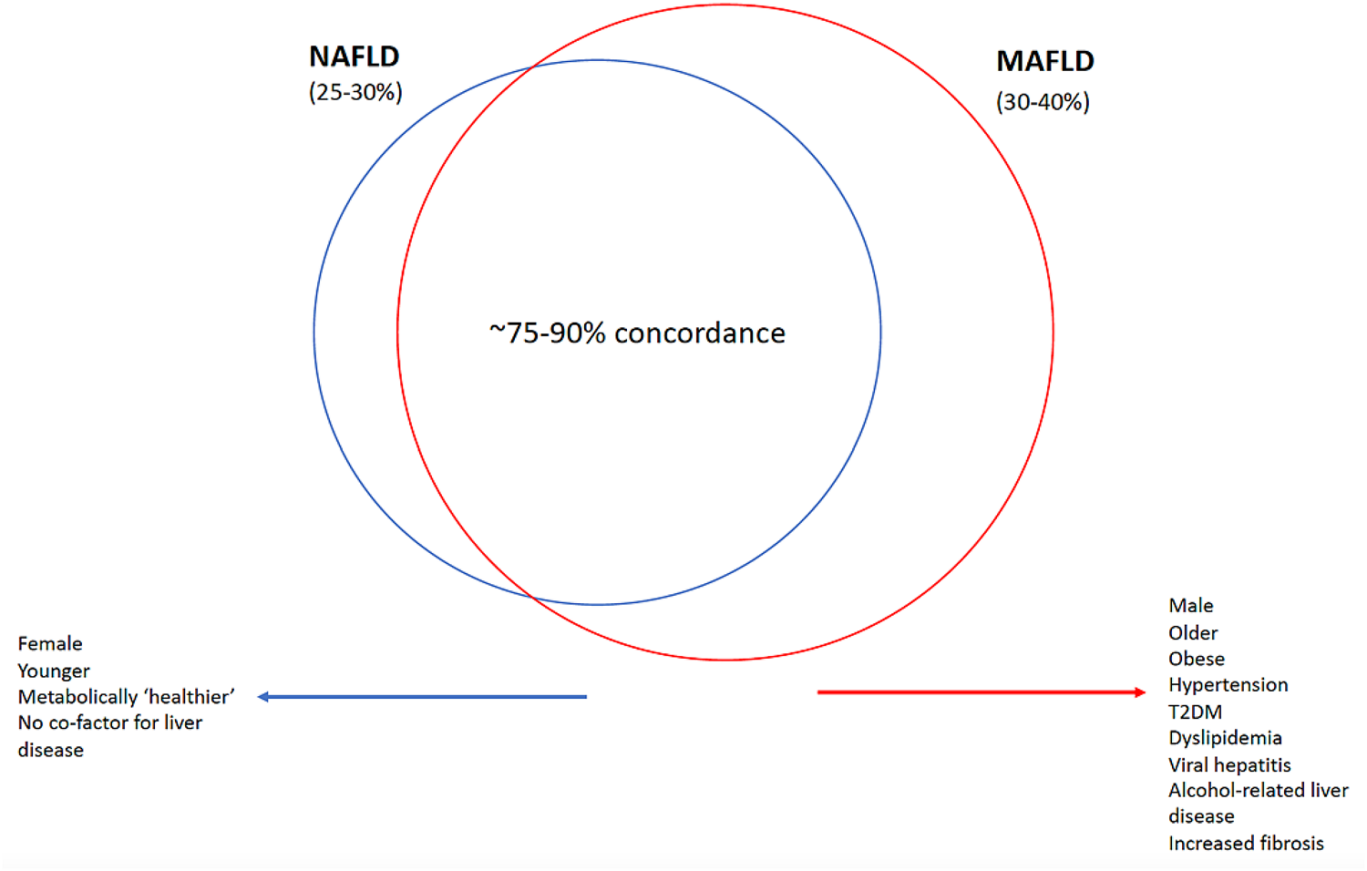
Differences in NAFLD and MAFLD contributing to prevalence difference

A uniform finding among studies was that in comparison to those meeting a diagnosis for NAFLD, those with MAFLD were more likely to be males (paralleling difference in excessive alcohol consumption between genders in these studies), have more participants with one or more components of metabolic syndrome, and a greater proportion with indeterminate or high-risk for fibrosis on basis of non-invasive tests (Fig. 1.). This is likely related to the inherently inclusive diagnostic criteria for MAFLD, requiring one or more clinical features of metabolic dysfunction to be present, as well as for allowing for co-existence of alternate etiology of liver disease, which may result in an additive or synergistic impact on fibrogenesis. This requires further evaluation in prospective studies. These differences are even more stark when comparing participants meeting the diagnosis for MAFLD without NAFLD compared to NAFLD without MAFLD, raising the prospect of greater granularity in risk stratifying patients with fatty liver disease, with a small proportion with potentially otherwise ‘metabolically healthy’ fatty liver. Apart from a minority (*n* = 5/35, 14%) [15, 16, 28, 44, 50], the epidemiologic studies consistently demonstrate that point prevalence is higher for MAFLD when redefined from NAFLD, which fits in with its inherently inclusive diagnostic criteria.

Another point to highlight is that the difference in prevalence of MAFLD and NAFLD is linked to the background prevalence of other causes of liver disease, with a greater difference in point prevalence in those populations in which alternate causes of liver disease are more prevalent. Once more this is related to the polarizing diagnostic criterion of co-factor for liver disease, which allows for existence of alternate etiologies of liver disease for MAFLD but not NAFLD (Table 1) [5, 7, 8].

## Natural history

Beyond epidemiology, a highly relevant aspect of the name change for clinicians is in determining any major differences in clinical outcomes. This allows a physician to appropriately counsel the patient on prognostication and focus therapeutic efforts in ameliorating this risk. Many researchers have made concerted efforts in establishing if the shift in nomenclature has resulted in a disease with heightened risk, particularly given the allowance for co-occurrence of alternate etiology of liver disease in the definition.

### Mortality

**Fig. 2:**
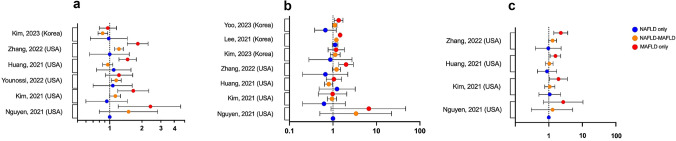
Forest plot of studies reporting hazard ratios for: **a** overall mortality; **b** cardiovascular disease-related mortality; **c** cancer-related mortality according to fatty liver disease diagnosis

*Overall:* Nguyen and colleagues trichotomized NHANES III participants into non-NAFLD-MAFLD, overlap NAFLD-MAFLD and non-MAFLD-NAFLD groups, and demonstrated a significant difference in 15-year all-cause mortality between the groups, 26.2% vs 21.1% vs 10.6% (*p* < 0.0001), respectively [22]. Those with MAFLD without NAFLD had a 2.4-fold increased risk for mortality compared to NAFLD without MAFLD on a model adjusted for demographic features, smoking status, viral hepatitis, fibrosis stage and weight (Table 3 and Fig. 2a). Older age, current or former smoking status, being African American and viral hepatitis were all independently associated with all-cause mortality on a multivariable regression model. Similarly, Kim et al. utilized the NHANES III database and stratified participants according to the three fatty liver groups per Nguyen et al. and reported on long-term outcome, with a median follow-up time of 23.2 years [55]. Once more, the authors demonstrated that those with MAFLD are at higher risk for all-cause mortality than those with NAFLD. First, participants with MAFLD had a 17% increased risk of all-cause mortality compared with those without MAFLD on a comprehensive multivariable Cox proportional model, while there was no difference between those with NAFLD compared to non-NAFLD (*p* = 0.35). Second, adjusting for the same demographic, lifestyle, clinical and laboratory covariates, only participants with NAFLD-MAFLD and non-NAFLD-MAFLD were determined to have a significantly increased risk for all-cause mortality compared with those without any steatosis (Table 3 and Fig. 2a); no difference was observed for those with non-MAFLD-NAFLD.

**Table 3:** Summary of studies reporting on hazard ratio for mortality according to fatty liver disease diagnosis

Study	Country	Follow-up period (years)	Mortality			
			Overall	CVD-related	Cancer-related	Liver-related
Nguyen et al. [22]	USA	15	MAFLD only: 2.4 (1.2–4.6) NAFLD-MAFLD: 1.5 (0.8–2.8) NAFLD only: reference	MAFLD only: 6.7 (0.9–47.1) NAFLD-MAFLD: 3.4 (0.5–22.3) NAFLD only: reference	MAFLD only: 2.7 (0.7–10.5) NAFLD-MAFLD: 1.3 (0.3–5.2) NAFLD only: reference	–
Kim et al. [55]	USA	Median 23.2 IQR 21.7–25.0	MAFLD only: 1.66 (1.19–2.32) NAFLD-MAFLD: 1.13 (1.00–1.26) NAFLD only: 0.94 (0.60–1.46) MAFLD vs non-MAFLD: 1.17 (1.04–1.32) NAFLD vs non-NAFLD: 1.05 (0.95–1.17)	MAFLD only: 0.98 (0.46–2.08) NAFLD-MAFLD: 0.95 (0.74–1.21) NAFLD only: 0.62 (0.20–1.92) MAFLD vs non-MAFLD: 0.95 (0.75–1.21) NAFLD vs non-NAFLD: 0.92 (0.71–1.17)	MAFLD only: 1.95 (1.05–3.62) NAFLD-MAFLD: 1.07 (0.76–1.52) NAFLD only: 1.07 (0.51–2.24) MAFLD vs non-MAFLD: 1.15 (0.82–1.62) NAFLD vs non-NAFLD: 1.02 (0.75–1.39)	–
Younossi et al. [56]	USA	Median 22.8 IQR 20.4–24.8	MAFLD only: 1.22 (0.91–1.64) NAFLD-MAFLD: 1.15 (1.04–1.28) NAFLD only: 1.07 (0.70–1.62)	–	–	–
Huang et al. [17]	USA	Median 22.8	MAFLD only: 1.47 (1.22–1.77) NAFLD-MAFLD: 0.96 (0.86–1.07) NAFLD only: 1.09 (0.75–1.58) MAFLD vs non-MAFLD: 1.03 (0.93–1.15) NAFLD vs non-NAFLD: 0.81 (0.66–1.00)	MAFLD only: 1.05 (0.70–1.58) NAFLD-MAFLD: 0.80 (0.64–0.98) NAFLD only: 1.24 (0.48–3.25) MAFLD vs non-MAFLD: 0.83 (0.68–1.02) NAFLD vs non-NAFLD: 0.80 (0.65–0.98)	MAFLD only 1.58 (1.09–2.28) NAFLD-MAFLD 1.04 (0.81–1.34) NAFLD only 0.89 (0.46–1.72) MAFLD vs non-MAFLD: 1.12 (0.88–1.41) NAFLD vs non-NAFLD: 0.96 (0.76–1.21)	–
Zhang et al. [57]	USA	Median 23.2 IQR 21.6–25.0	MAFLD only: 1.83 (1.46–2.28) NAFLD-MAFLD: 1.22 (1.11–1.34) NAFLD only: 1.00 (0.65–1.52)	MAFLD only: 2.00 (1.36–2.94) NAFLD-MAFLD: 1.21 (0.97–1.49) NAFLD only: 0.67 (0.20–2.20)	MAFLD only: 2.29 (1.42–3.69) NAFLD-MAFLD: 1.30 (1.00–1.70) NAFLD only: 0.97 (0.40–2.33)	–
Moon et al. [58]	Korea	Median 15.7 IQR 13.9–15.9	MAFLD vs non-MAFLD: 1.36 (1.08–1.73) NAFLD vs non-NAFLD: 1.20 (0.94–1.53)	MAFLD vs non-MAFLD: 0.99 (0.55–1.78)	MAFLD vs non-MAFLD: 1.48 (0.98–2.23)	MAFLD vs non-MAFLD: 2.76 (1.07–7.13)
Kim et al. [43]	Korea	Median 5.7	MAFLD only: 0.96 (0.80–1.16) NAFLD-MAFLD: 0.86 (0.78–0.96) NAFLD only: 0.98 (0.66–1.46)	MAFLD only: 1.18 (0.77–1.83) NAFLD-MAFLD: 1.13 (0.87–1.46) NAFLD only: 0.87 (0.28–2.74)	–	–
Lee et al. [39]	Korea	Median 10.1	–	MAFLD only: 1.46 (1.41–1.52) NAFLD-MAFLD: 1.20 (1.17–1.24) NAFLD only: 1.12 (0.96–1.30)	–	–
Yoo et al. [45]	Korea	Median 8.8	–	MAFLD only: 1.35 (1.07–1.70) NAFLD-MAFLD: 1.10 (0.97–1.24) NAFLD only: 0.67 (0.38–1.19) MAFLD vs non-MAFLD: 1.14 (1.02–1.28) NAFLD vs non-NAFLD: 1.07 (0.95–1.21)	–	–

Mortality data presented as hazard ratio (95% confidence interval)

Fully adjusted model presented from each study when available

Unless explicitly stated, the reference group for each hazard ratio is in those without hepatosteatosis

*CVD* cardiovascular disease, *USA* United States of America, *MAFLD* metabolic-associated fatty liver disease, *NAFLD* non-alcoholic fatty liver disease, *IQR* interquartile range

It is noteworthy that a single study also reporting from NHANES III did not demonstrate a difference in all-cause mortality in those with MAFLD or NAFLD, when compared to non-MAFLD/NAFLD participants [56]. Although alcohol-related liver disease was accounted for in this model, the definition was based on a lower threshold for alcohol consumption (≥ 20 g/day in males; ≥ 10 g/day in females) than the other two studies reporting from NHANES III (≥ 30 g/day in males; ≥ 20 g/day in females), which might have influenced the findings. There were other differences between the covariates included in respective models, which might also have led to a disparity in findings. Finally, in Huang et al. study [17] also utilizing NHANES, MAFLD but not NAFLD was determined to increase risk of overall mortality compared with non-fatty liver disease participants, except when the model was adjusted for metabolic risk factors, suggesting it is these risk factors which associate with heightened mortality risk.

In the report by Wang et al. from the Kailuan Study in China following over 150,000 participants for a median of 12.7 years, the annual all-cause mortality rate of MAFLD was higher than NAFLD for all age groups and between genders [29], consistent with NHANES III literature. Furthermore, on subgroup analysis, meeting the MAFLD criteria according to T2DM or metabolic dysfunction had a more profound negative impact on mortality than overweight/obesity criterion (HR 1.41 [95% CI 1.18–1.67] up to HR 4.26 [95% CI 1.74–10.43] vs HR 0.32 [95% CI 0.15–0.57] up to HR 1.06 [0.64–1.74] in various age-/gender-stratified groups); the cumulative number of criteria met led to higher risk of mortality (HR up to 4.26 [95% CI 1.74–10.43] for one criteria met vs HR up to 11.40 [95% CI 2.69–48.35] for two or three criteria met); and presence of an additional cause of liver disease (viral and/or alcohol-related) compounded the risk of mortality (HR up to 1.77 [95% CI 1.27–2.48] without additional liver disease vs up to 9.86 [95% CI 2.44–39.98] with co-existent liver disease). This concept of a difference in outcome according to MAFLD criteria met was also reported from NHANES III, again with T2DM and metabolic dysfunction presenting a greater risk than MAFLD with overweight/obesity alone [57].

Two studies originating from different cohorts in Korea have reported on the comparison in mortality between NAFLD and MAFLD. One study including adults aged 40–70 years once more demonstrated the impact of a name change has on mortality, with MAFLD conferring a 36% higher risk of all-cause mortality after adjustment for relevant covariates, while there was no difference in mortality in NAFLD participants with the same regression model (Table 3) [58]. The adverse impact of MAFLD on mortality remained even after adjusting for viral hepatitis and excess alcohol consumption (HR 1.33, 95% CI 1.05–1.69). In another study reporting from the large cross-sectional Kangbuk Samsung Health Study, enrolling all adults 18 years and older between 2002 and 2012 and followed for a median of 5.7 years, all-cause mortality was higher in MAFLD participants than those without MAFLD (log-rank *p*-value < 0.001 from Kaplan–Meier curve) [43], with insignificant difference between NAFLD vs non-NAFLD (*p* = 0.20). There was delineation in survival curves for groups stratified as non-MAFLD-NAFLD, NAFLD-MAFLD and non-NAFLD-MAFLD (log-rank *p* < 0.001), with the MAFLD-only group at highest risk of mortality (HR 1.67, 95% CI 1.40–1.99) and the NAFLD-only group at no higher risk of mortality than non-steatotic participants (HR 0.84, 95% CI 0.57–1.23) on unadjusted models, with the statistical significance dissipating in the MAFLD-only group on multivariable models (Table 3 and Fig. 1a).

*Cardiovascular disease and cancer-related:* A strength of the study by Younossi et al. was in reporting on cause-specific mortality data, with CVD-related death most common cause of mortality in MAFLD participants (34.5%) followed by extra-hepatic malignancy (20.4%), both outnumbering liver-related death (6.7%) during the 20-year follow-up period [56]. All the authors reporting from NHANES III were consistent in reporting CVD as the foremost cause of death, closely followed by cancer [22, 55, 57]. Nguyen and colleagues demonstrated that the mortality difference between MAFLD-only, NAFLD-MAFLD and NAFLD-only groups persisted for cardiovascular disease (CVD)-related mortality (*p* = 0.009) and non-cancer/non-CVD-related mortality (*p* = 0.002) but not cancer-related mortality (*p* = 0.2) [22].

On sensitivity analysis of cause-specific mortality, Kim et al. established there was no difference in CVD-related mortality and cancer-related mortality in the fully-adjusted model between MAFLD vs non-MAFLD (*p* = 0.69 and *p* = 0.41, respectively) and NAFLD vs non-NAFLD (*p* = 0.48 and *p* = 0.89, respectively) [55]. However, non-NAFLD-MAFLD conferred a heightened risk for cancer-related mortality on the complete model, with non-steatotic participants as the reference (Table 3 and Fig. 2c). This may suggest that co-factor for liver disease may be a determinant for increased risk of carcinogenesis, although there was no granular data on specific malignancies to draw conclusions about where this risk may lie (i.e., hepatic vs extra-hepatic).

Similarly, Moon et al. found no difference in CVD-related mortality between those with and without MAFLD (*p*-value 0.66–0.98 on all adjusted models), but once more MAFLD portended a higher cancer-related mortality (HR 1.52 [95% CI 1.01–2.30] up to HR 1.63 [95% CI 1.13–2.36] on adjusted models), except for when the model was adjusted for viral hepatitis and excess alcohol consumption (Table 3) [58]. This adds further weight to the hypothesis that cancer-related mortality in MAFLD may account for by the synergistic carcinogenesis occurring with additional liver disease. Contrary to Kim et al. the authors of this study were able to reveal cause-specific cancer deaths, with ‘other’ unspecified cancer (51.0%) far outweighing liver (18.4%) and lung (16.3%) as the most common cause of cancer-related death [58].

In another Korean study by Kim and colleagues with shorter duration of follow-up, CVD-related mortality was higher in MAFLD participants than those without MAFLD (log-rank *p*-value < 0.001 from Kaplan–Meier curve) as well as NAFLD vs non-NAFLD (*p* = 0.002) [43], while those by Lee et al. [39] and Yoo et al. [45] demonstrate an apparent stepwise hierarchy in risk stratification for CVD mortality in fatty liver disease with the advent of the nomenclature change; highest for isolated MAFLD, followed by concordant fatty liver disease, and the least risk—with near equipoise to the general non-fatty liver disease controls—in those with NAFLD-alone (Table 3).

*Liver-related:* Death from liver disease was not well reported among studies, with authors utilizing NHANES III raising difficulties with accessing this linked data due to the few numbers of liver-related deaths [22, 55, 57]. However, Younossi et al. were able to access this specific data and reported liver-related mortality was higher for MAFLD than NAFLD (3.01%, 95% CI 1.99–4.03 vs 1.81%, 95% CI 0.95–2.66), and although the influence of various covariates on CVD-related and extra-hepatic malignancy-related mortality was similar between the two groups, it was markedly different for liver-related mortality [56]. Whereas high-risk for fibrosis was the greatest influence over liver-related mortality in both MAFLD and NAFLD (HR 17.15, 95% CI 4.55–64.65 and HR 9.26, 95% CI 1.84–46.33, respectively), the other covariates with most influence were alcohol-related liver disease (HR 4.50, 95% CI 1.89–10.75) and chronic kidney disease (HR 2.92, 95% CI 1.21–7.01) for MAFLD, while they were high C-reactive protein (CRP) (HR 4.47, 95% CI 1.35–14.77) and insulin resistance (HR 3.57, 95% CI 1.35–9.42) for NAFLD. It is vital to point out the differences in definitions between MAFLD and NAFLD once again, with the latter not allowing for inclusion of excessive alcohol consumption. As such, by definition, excessive alcohol consumption cannot be a predictor for liver-related outcome in NAFLD and by extension, this would impact how other covariates interact between the disease and outcome.

In the Korean study by Moon and colleagues, MAFLD was predictive for liver-related mortality even after comprehensive multivariable analysis accounting for demographic variables, comorbidities and high-sensitivity CRP (Table 3) [58].

**Fig. 3:**
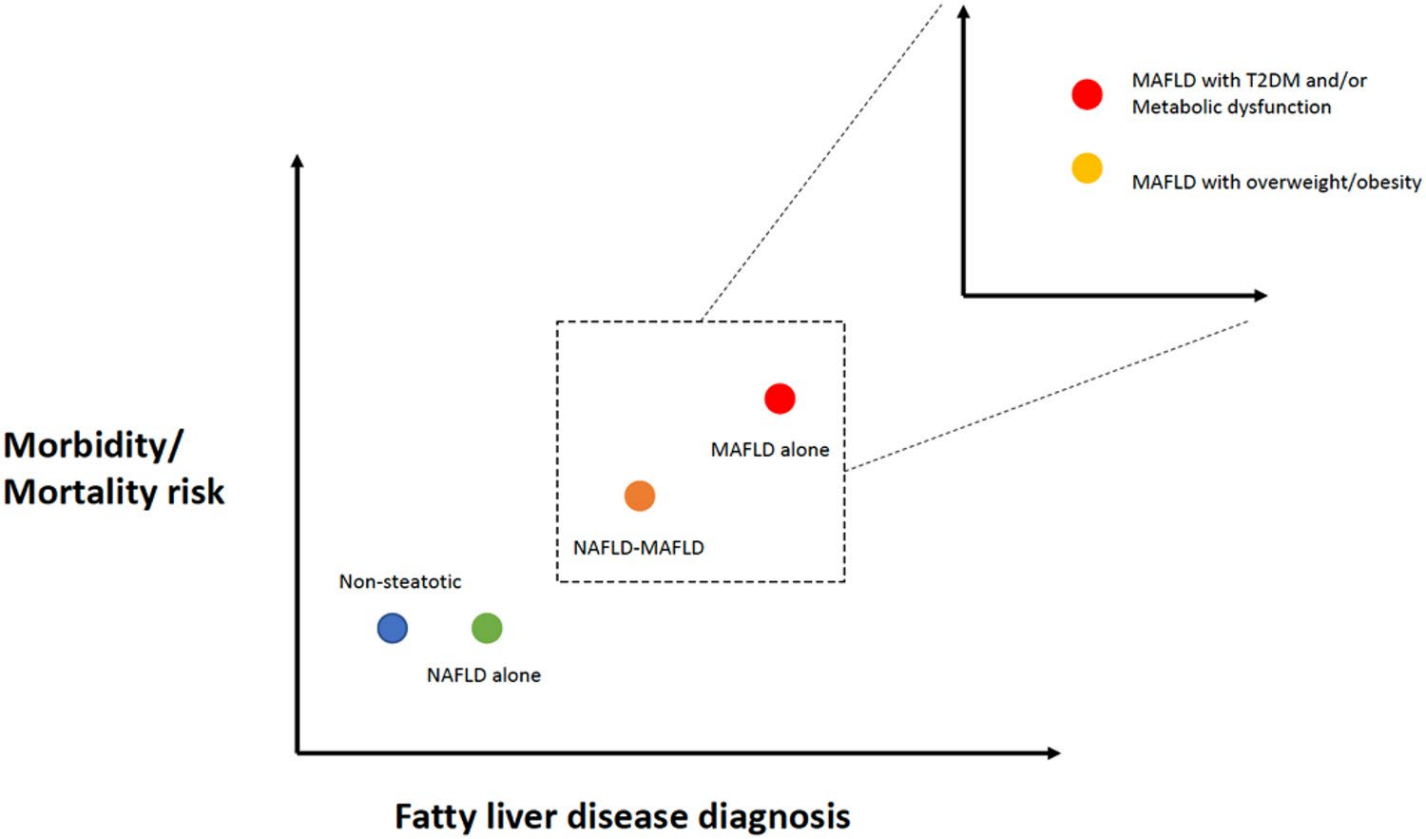
Schematic for difference in all-cause mortality between different forms of fatty liver disease

To surmise, these studies suggest that meeting the diagnostic criteria for MAFLD is more hazardous than a diagnosis of NAFLD, with a higher risk of mortality (in particular, all-cause and liver-related), MAFLD without NAFLD (i.e., fatty liver in the presence of co-factor for liver disease) leads to a compounded risk of death, fulfilling different criterion for MAFLD may impact mortality risk (with highest risk for those meeting T2DM and metabolic dysfunction criteria) and that there is a small cohort of fatty liver patients in the community who are metabolically ‘healthier’ that do not appear to have adverse outcomes compared to non-steatotic ‘healthy’ participants (Figs. 2 and 3.). The reported differential mortality risk between NAFLD and MAFLD may be in part resultant from the multivariate models utilized to adjust for risk (e.g., not being able to adjust for relevant metabolic covariates given these are contained within the MAFLD diagnostic criteria) or due to the inclusive nature of the MAFLD diagnostic criteria, allowing for co-existence of alternate etiologies of liver disease, which as discussed earlier may have a deleterious and compounding impact on mortality. This has implications for public health and research efforts as it stratifies the ballooning problem of fatty liver into at-risk groups for which targeted interventions are most needed.

### Morbidity

The literature describing the differential morbidity between NAFLD and MAFLD is less certain and mature than mortality data. While some authors report a higher incidence of general and central obesity [52], T2DM [52] and CVD [39, 52] in MAFLD compared with NAFLD, others have found an increased morbidity from extra-hepatic disease in MAFLD and NAFLD compared to non-fatty liver participants, but no difference between the two conditions [28, 30, 42, 59]. However, this area of research is still in its infancy and the volume of studies is limited in comparison to those conducted in the NAFLD-alone arena, which have allowed for numerous meta-analyses in individual outcomes [60–67].

Scarce literature has examined the impact of the change in nomenclature on malignancy risk, whether primary hepatic or extra-hepatic. A single Korean study examining over 10 million adults aged between 40 and 64 years demonstrated that the risk for incident colorectal cancer was highest among those with isolated MAFLD (HR 1.32, 95% CI 1.28–1.35), followed by those with NAFLD-MAFLD (HR 1.18, 95% CI 1.16–1.20), and last in those with non-MAFLD-NAFLD (HR 1.16, 95% CI 1.06–1.28) [68]. A study by Yuan and colleagues [35] examining a large cohort from China followed for a median duration of over 12 years reported contrary results regarding extra-hepatic malignancy with NAFLD-MAFLD carrying an increased risk for colorectal cancer (HR 1.19, 95% CI 1.00–1.41), thyroid cancer (HR 1.62, 95% CI 1.11–2.35), renal cancer (HR 1.58, 95% CI 1.19–2.09), prostate cancer (HR 1.48, 95% CI 1.04–2.11) and breast cancer (HR 1.29, 95% CI 1.02–1.64), but MAFLD alone not increasing the risk for any of the twelve malignancies investigated. However, these models adjusted for excessive alcohol consumption, with the authors also demonstrating that those with MAFLD and excessive alcohol consumption had a higher risk of developing extra-hepatic malignancy (HR 1.14, 95% CI 1.01–1.29), which was not seen in those with MAFLD and viral hepatitis (HR 1.17, 95% CI 0.83–1.65) or MAFLD without co-factor for liver disease (HR 1.03, 95% CI 0.97–1.10).

Only a single study has reported on incidence of hepatocellular carcinoma (HCC) in those with NAFLD compared to MAFLD. In this retrospective study from Geneva examining HCC incidence between 1999 and 2014, the MAFLD-HCC age-standardized incidence rose from 1.30 (95% CI 0.75–2.10) to 5.03 (95% CI 4.01–6.23) per 100,000, with a fivefold higher age-standardized incidence than NAFLD-HCC in males and twofold higher age-standardized incidence in females in 2014 [69].

## Future directions

Although the debate surrounding the nomenclature shift may rage on, researchers continue to examine how the diagnostic criteria have impacted epidemiology and natural history of fatty liver disease. Yet more is to be learnt over the coming years and decades. We recommend the following priorities in studies reporting on the epidemiology and clinical outcomes of fatty liver disease:

1. Prevalence studies originating beyond the US and Asia, to better establish if there is geographic variation for prevalence between these two conditions and the factors contributing to these differences (environmental vs genetic vs other)
2. Exploring differences in liver-related outcome, CVD-related outcome and incidence of hepatic and extra-hepatic malignancy between NAFLD and MAFLD
3. Evaluating the morbidity and mortality of MAFLD across gender and different age groups, including older persons, to determine whether there are age- or gender-specific differences in clinical outcomes over time
4. Investigating differences in outcome according to the specific MAFLD criteria being met (overweight/obesity vs T2DM vs metabolic dysfunction, as well as general vs central obesity), number of MAFLD criteria met (1 vs 2 vs 3), and in relation to presence or absence of co-factor for liver disease (particularly the impact co-occurrence of viral and alcohol-related liver disease have on hepatic and extra-hepatic malignancy)
5. Determining how diet and lifestyle, including participation in physical activity, influence outcome in MAFLD compared to NAFLD

## Conclusions

The dawn of MAFLD has led to an increased prevalence of fatty liver disease, with a heightened risk for overall mortality. However, much is still to be established about the impact of the name change, particularly on non-fatal clinical outcomes including CVD, liver decompensation and malignancy.

### Supplementary Information

Below is the link to the electronic supplementary material.

Supplementary file1 (DOCX 337 KB)

## Data Availability

This is a review article utilising data from other authors/published papers and, as such, data availability statement not required.
