# Supplementary material for: Current understanding and future perspectives on the impact of changing NAFLD to MAFLD on global epidemiology and clinical outcomes
Source: Hepatol Int. 2023 Aug 9;17(5):1082–97. doi: 10.1007/s12072-023-10568-z (PMC10522780; doi:10.1007/s12072-023-10568-z)
Supplement: Supplementary file 1 — Supplementary file1 (DOCX 337 KB) [file 12072_2023_10568_MOESM1_ESM.docx]

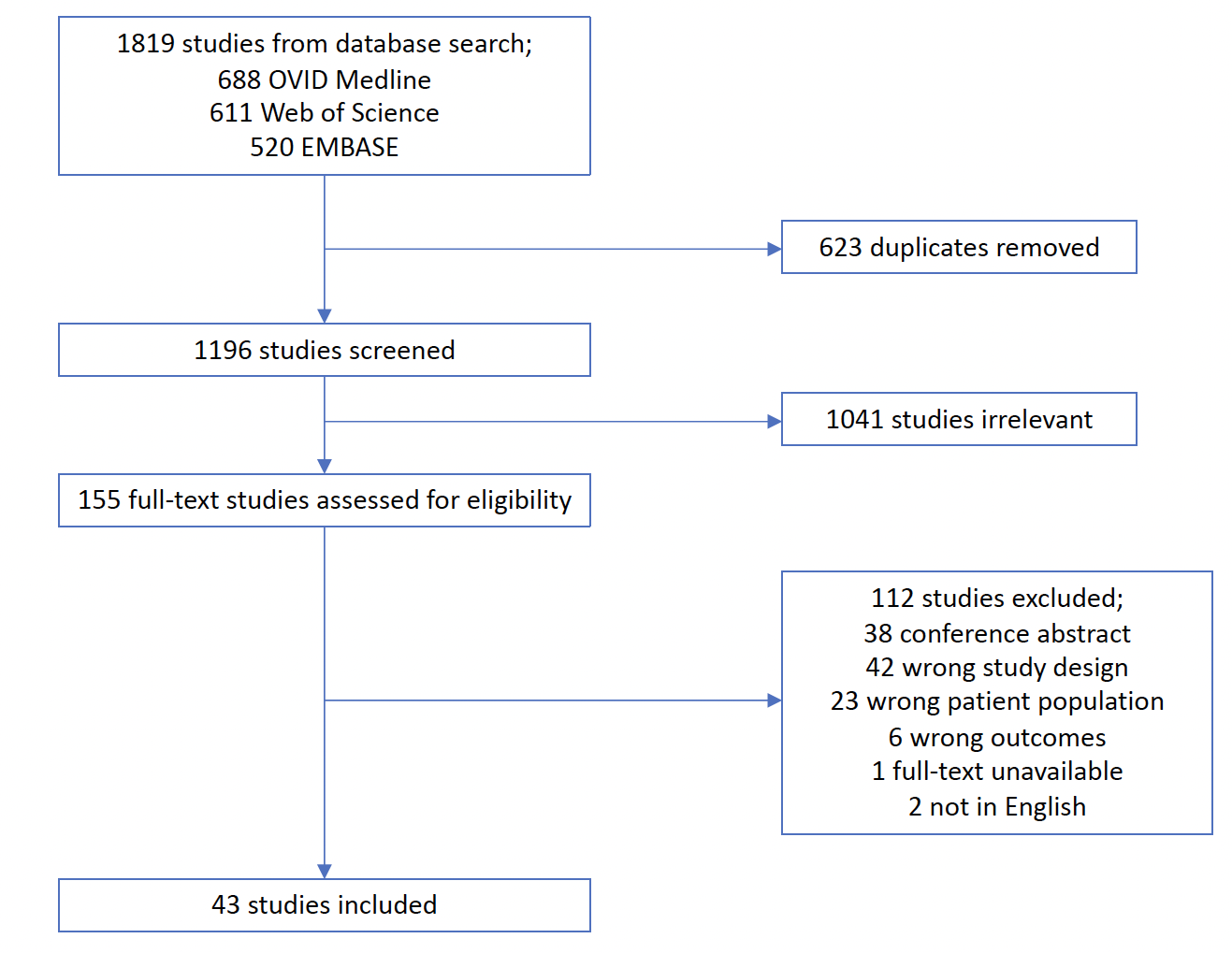


**Supplementary Figure 1.** PRISMA flowchart demonstrating the search and selection process of studies within systematic review
